# Multiple DNA repair pathways contribute to MMS-induced post-replicative DNA synthesis in *S. pombe* .

**DOI:** 10.17912/micropub.biology.000974

**Published:** 2023-10-02

**Authors:** Seong Min Kim, Susan L. Forsburg

**Affiliations:** 1 Molecular and Computational Biology, University of Southern California, Los Angeles, California, United States; 2 University of Southern California, Los Angeles, California, United States

## Abstract

Replication stress can induce DNA synthesis outside of replicative S-phase. We have previously demonstrated that fission yeast cells stimulate DNA synthesis in G2-phase but not in M-phase in response to DNA alkylating agent MMS. In this study, we show that various DNA repair pathways, including translesion synthesis and break-induced replication contribute to post-replicative DNA synthesis. Checkpoint kinases, various repair and resection proteins, and multiple polymerases are also involved.

**Figure 1. DNA repair pathways, checkpoints, and polymerases are involved in MMS-induced post-replicative DNA synthesis.**
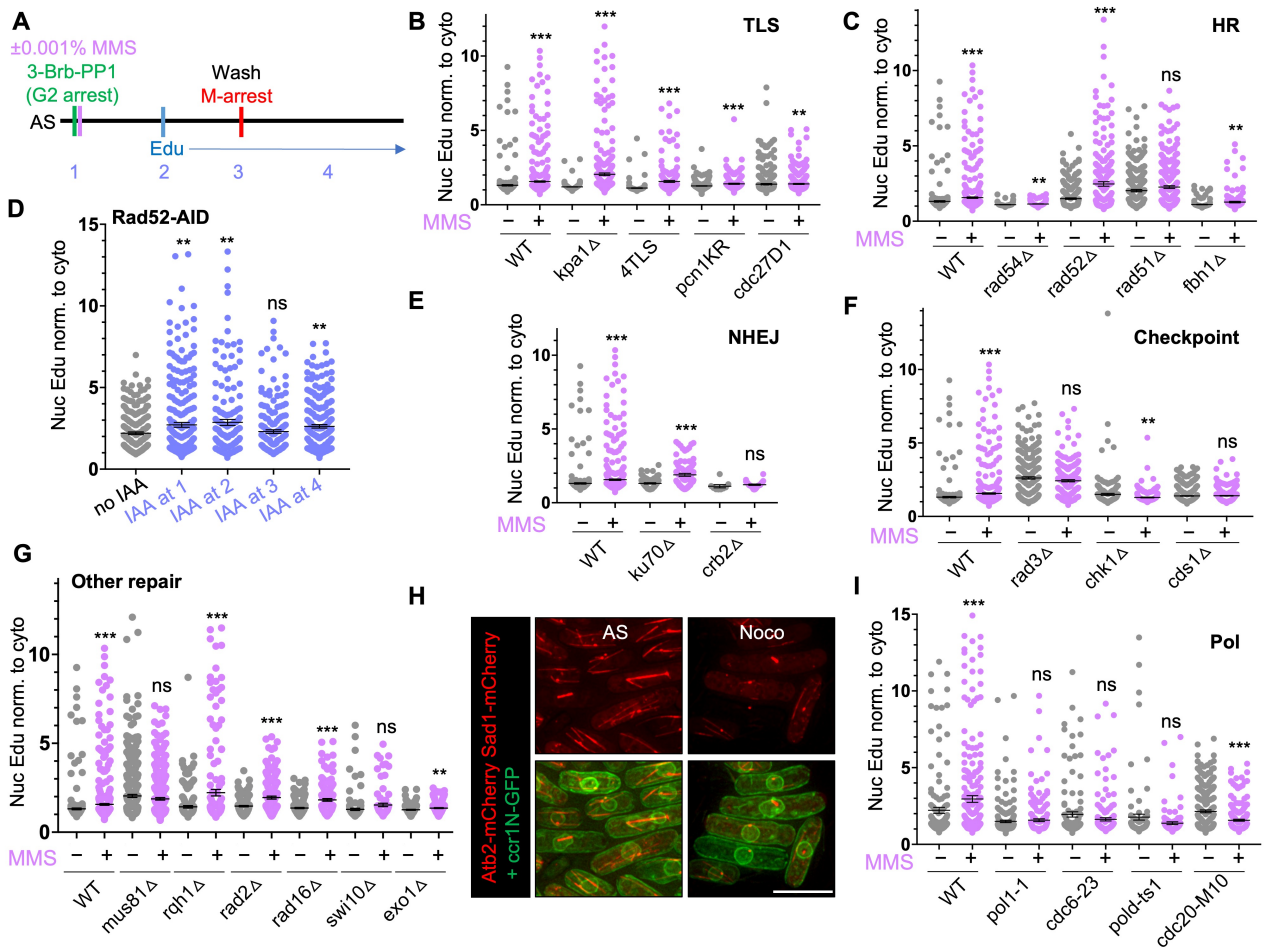
( **A** ) Experimental procedure outline as detailed in Method section. Cells with *cdc2-asM17 cut9-665* background were arrested in G2 by 3-Brb-PP1 in the presence or absence of 0.001% MMS. 5-Ethynyl-2’-deoxyuridine (EdU) was added to cells with during G2 arrest and during release to 36 ºC for M-arrest. AS indicates asynchronous culture. Numbers 1-4 indicate when auxin (IAA) was added for panel (D). 1: IAA added to the asynchronous culture with 3-Brb-PP1, 1.5 h before EdU. 2: IAA added at the same time as EdU. 3: IAA added just before the shift to 36 ºC for M-arrest. 4: IAA added 1.5 h after shift to 36 ºC. ( **B,C, E-G** ) Quantification of nuclear EdU intensity normalized to cytoplasmic intensity in cells treated as in (A), showing wildtype (WT) and mutant strains involved in ( **B** ) translesion synthesis (TLS), ( **C** ) homologous recombination (HR), ( **E** ) non-homologous end joining (NHEJ), ( **F** ) checkpoint kinases, ( **G** ) various DNA repair pathways. ( **D** ) Quantification of normalized nuclear EdU intensity in G2-arrested *Rad52-AID cdc2-asM17 cut9-665* cells. IAA was added at the beginning of G2-arrest (1) , during G2-arrest (2), at the beginning of M-arrest (3), or during M-arrest (4). ( **H** ) Images of spindle fiber (Atb2-mCherry), spindle pole body (Sad1-mCherry) (top) and nuclear membrane marker Ccr1N-GFP (ccr1(275-678)-GFP)) (bottom) in asynchronous culture (AS), or after M-arrest via nocodazole (Noco). Scale bar: 10 µm. ( **I** ) WT and temperature-sensitive mutant strains with *cdc2-asM17 nda3-KM311* background treated as in (A) but at 36 ºC and using nocodazole for M-arrest. All EdU quantification is done with N > 100 cells.

## Description

Various studies in mammalian cells have shown that replication stress can drive DNA synthesis outside of replicative S-phase defined as Mitotic DNA Synthesis (MiDAS) (Minocherhomji et al. 2015; Bhowmick et al. 2016; Lezaja et al. 2021; Wu et al. 2023) . Studies done in budding yeast (Ivanova et al. 2020) and *C. elegans* (Sonneville et al. 2019) have demonstrated that post-replicative DNA synthesis occurs in lower eukaryotes as well. Replisome maintenance (Sonneville et al. 2019) , replication structure processing (Minocherhomji et al. 2015; Calzetta et al. 2020; Garribba et al. 2020) , and DNA damage repair (Minocherhomji et al. 2015; Bhowmick et al. 2016; Wu et al. 2023) are some of the processes involved in MiDAS.

DNA synthesis induced by replication inhibitor aphidicolin continues throughout G2 phase in mammalian cells (Mocanu et al. 2022) and fission yeast *Schizosaccharomyces pombe* have low amount of DNA synthesis in post-replicative G2 (Kelly and Callegari 2019) . These findings indicate that post-replicative DNA synthesis is not limited to mitotic phase. We have also recently demonstrated that replication stress induced by DNA alkylating agent methyl methanesulfonate (MMS) stimulate DNA synthesis during G2 but not during mitosis in *S. pombe* (Kim and Forsburg 2023) . In this study, we investigated what pathways and proteins contribute to MMS-induced post-replicative DNA synthesis.

As before (Kim and Forsburg 2023) , we used cells that have the analogue-sensitive *cdc2-asM17* allele that enables G2-arrest with ATP analog 3-Brb-PP1 ((3-[(3-bromophenyl)methyl]-1-(1,1-dimethylethyl)-1H-pyrazolo[3,4-d]pyrimidin-4-amine) (Aoi et al. 2014; Singh et al. 2021) and the *cut9-665* temperature-sensitive allele that arrests cells in mitosis when placed at 36 ºC (Samejima and Yanagida 1994) . Our previous work demonstrated that both *cdc2-asM17* and *cut9-665* alleles uniformly arrest cells in G2 and mitotic phase, respectively (Kim and Forsburg 2023) . The strains were engineered to take up the thymidine analogue EdU (5-Ethynyl-2’-deoxyuridine) for detection of DNA synthesis (Hodson et al. 2003) . EdU was added during G2-arrest and was maintained through G2- and mitotic arrest ( Figure 1A ). Replication stress was induced by treatment of 0.001% MMS, at a concentration that does not perturb cell cycle (Kim and Forsburg 2023) . After Click-it reaction, nuclear EdU signal intensity was measured and normalized to cytoplasmic signal for quantification. As mutant strains can have a cell cycle profile that differs from its counterpart wildtype (WT), comparisons were made between untreated and MMS-treated conditions within the strain ( Figure 1B,C, E -G, I).

We have previously shown that most of post-replicative DNA synthesis induced by MMS is occurring during G2 not M (Kim and Forsburg 2023) . However, as EdU was present during G2 and M arrest, we will refer to the post-replicative DNA synthesis as occurring in G2/M. Replication stress induced by MMS increases EdU intensity in WT in post-replicative G2/M ( Figure 1B ) (Kim and Forsburg 2023) . We set out to identify which DNA repair pathway proteins contribute to MMS-stimulated DNA synthesis.

Translesion synthesis (TLS) is one of the major repair pathways eukaryotic cells rely on to bypass replication blocks (rev. in (Powers and Washington 2018; Maiorano et al. 2021) ). Cells deficient in Kpa1, the translesion DNA repair polymerase kappa, had little nuclear EdU levels in untreated condition ( Figure 1B ). MMS treatment significantly increased EdU levels in *kpa1* Δ cells, indicating that Kpa1-deficiency is not sufficient to reduce MMS-stimulated post-replicative DNA synthesis. However, when cells were deficient in other TLS proteins as well (4TLS: *kpa1* Δ *, rev1* Δ *, rev3* Δ *, polηΔ (eso1-rad30Δ)* ), MMS treatment resulted in smaller increase in EdU levels. Proliferating cell nuclear antigen PCNA mutant *pcn1-K164R* that cannot be ubiquitinated and thereby fail to promote TLS (Frampton et al. 2006; Coulon et al. 2010) also had reduced G2/M DNA synthesis in both untreated and MMS-treated conditions. These results suggest that TLS pathway contributes to increased post-replicative DNA synthesis under replication stress and that multiple TLS proteins are likely involved in the process.

Cdc27-D1 is a polymerase δ C-terminus mutant that is deficient in break-induced replication (BIR), another important repair process at broken replication forks ( (Kraus et al. 2001; Tanaka et al. 2004) ). Cdc27-D1 mutant had comparable level of EdU intensity as WT in untreated condition but fail to further increase nuclear EdU levels in MMS condition ( Figure 1B ), indicating *cdc27-D1* suppressed increased DNA synthesis induced by MMS. This suggests that BIR pathway, along with TLS, contributes to replication-stress induced DNA synthesis in G2/M.

To test if homologous recombination (HR) repair pathway also contributes to increased EdU uptake during G2, we tested *rad54* Δ *,* *rad52* Δ, *rad51* Δ, and *fbh1* Δ strains ( Figure 1C ). Rad54 is the motor protein that translocates along dsDNA during HR; Rad52 promotes the displacement of RPA by Rad51 which then forms nucleoprotein filaments for homologous sequence search (rev. in (Li and Heyer 2008) ). Fbh1 is a F-box DNA helicase which is frequently found to be deleted in *rad52* Δ as critical functions of Rad52 in HR repair are circumvented in the absence of Fbh1 (Osman et al. 2005) . Interestingly, *rad54* Δ and *fbh1* Δ strains had reduced EdU uptake in both untreated and MMS -treated conditions while *rad52* Δ and *rad51* Δ showed similar or higher levels of EdU than WT. MMS treatment induced significant increase in *rad52* Δ while no further increase was observed in *rad51* Δ cells. These results suggest that functions of Rad54 and Fbh1 that are not directly related to HR repair may be playing a role in stimulating EdU uptake during G2/M.

Chronic Rad52 deficiency frequently results in the loss of Fbh1 as critical functions of Rad52 in recombination is opposed by Fbh1 (Osman et al. 2005; Lorenz et al. 2009) . To test whether the increased amount of DNA synthesis observed in *rad52∆* cells is consequential to Fbh1 loss, we used an auxin-inducible degron system of Rad52 (Watson et al. 2021) . In this system, Rad52 protein is degraded within 15 minute after 100 nM auxin (5’adamantyl-IAA, IAA) treatment (Watson et al. 2021) . This acute degradation of Rad52 circumvent Fbh1 loss that may arise in permanently Rad52-deficient cells. IAA added at the beginning and during G2-arrest (1,2 in Figure 1A,D ) and during M-arrest (4 in Figure 1A,D ) significantly increased nuclear EdU levels ( Figure 1D ), indicating loss of Rad52 increases post-replicative DNA synthesis, independently of Fbh1 loss. As acute Rad52 degradation resulted in greater increase in nuclear EdU levels compared to untreated *rad52∆* cells (which likely contain *fbh1∆* suppressor) and as *fbh1∆* cells had low EdU levels ( Figure 1C, 1D), Rad52 and Fbh1 likely have opposing roles in stimulating post-replicative DNA synthesis.

We next tested proteins involved in non-homologous end joining (NHEJ), another major DNA repair pathway (Chang et al. 2017) . Ku70 is a subunit of Ku protein that binds DNA ends during NHEJ (Zahid et al. 2021) . MMS-treated *ku70* Δ cells had greater nuclear EdU intensity than untreated cells, indicating Ku70 is not likely involved in MMS-induced DNA synthesis ( Figure 1E ). Crb2, a homolog of human 53BP1, is another DNA end binding protein that is associated with specific histone modifications (Hsiao and Mizzen 2013) . In addition to its role in checkpoint activation (Sofueva et al. 2010; Qu et al. 2012) , Crb2 plays a role in favoring NHEJ over HR by opposing excessive break resection (Leland et al. 2018) . Crb2 deficiency prevented post-replicative DNA synthesis in both untreated and MMS-treated cells ( Figure 1E ). This result suggest that Crb2 has a critical role in inducing DNA synthesis outside of S-phase. Crb2 plays a role in both checkpoint activation and NHEJ, but as Ku70 that has a critical function in NHEJ has little effect on MMS-induced DNA synthesis, it is likely that the checkpoint activating role of Crb2 is responsible for stimulating post-replicative DNA synthesis.

Therefore, we next examined to see if other checkpoint kinases are also involved in increased DNA synthesis in G2/M. Indeed, both the DNA damage response checkpoint kinase Chk1 and the replication stress response checkpoint kinase Cds1 contributed to G2/M DNA synthesis in untreated and in MMS-treated conditions ( Figure 1F ). However, ATR checkpoint kinase Rad3 appears to oppose DNA synthesis during G2/M. Rad3-deficient cells had much higher nuclear EdU levels compared to WT in both untreated and MMS-treated conditions ( Figure 1F ). MMS did not further increase EdU levels compared to its own untreated conditions. This suggests that Rad3 plays a role in keeping extraneous DNA synthesis in check while other checkpoint kinases contribute to maintaining post-replicative DNA synthesis.

Proteins that process replication structures and other DNA repair proteins were also tested ( Figure 1G ). Mus81 is a structure-specific endonuclease that plays a critical role in resolving replication and recombination intermediates (rev. in (Kim and Forsburg 2018) ). Mus81 deficiency had higher EdU levels compared to WT but failed to increase EdU levels in MMS treatment. This suggests that Mus81 plays a role in stimulating post-replicative DNA synthesis in response to replication stress or DNA damage resulting from the replication stress. In mammalian cells, RecQ DNA helicase RECQ5 has been shown to stimulate Mus81 and promote MiDAS (Di Marco et al. 2017) . Therefore, we tested whether fission yeast RecQ type DNA helicase Rqh1 plays a role in DNA synthesis in G2/M. Unlike mammalian cells, Rqh1-deficient fission yeast cells did not have a problem increasing EdU levels upon MMS treatment, indicating Rqh1 is dispensable for post-replicative DNA synthesis.

Rad2 is a FEN-1 endonuclease involved in nucleotide excision repair (Habraken et al. 1993) . Rad16-Swi10 (XPF-ERCC1 in human cells) are structure-specific endonucleases (SSE) that are also involved in nucleotide excision repair (Carr et al. 1994; Rödel et al. 1997). Exo1 is a double-stranded DNA 5'-3' exonuclease involved in long range resection (Szankasi and Smith 1995; Zhao et al. 2020) . MMS induced significant increases in EdU levels in *rad2* Δ and *rad16* Δ cells ( Figure 1G ), suggesting Rad2 and Rad16 are not likely to play a role in MMS-induced post-replicative DNA synthesis. MMS failed to induce increase in DNA synthesis in cells deficient in Swi10 or Exo1, suggesting that Rad16-independent role of Swi10 and Exo1 resection contribute to MMS-stimulated DNA synthesis in G2/M.

Lastly, we investigated which polymerases are involved in inducing post-replicative DNA synthesis in response to MMS treatment. We tested 4 temperature-sensitive polymerase mutant strains with *cdc2-asM17 nda3-KM311* background, using nocodazole for M-arrest. To confirm M-arrest by nocodazole, we imaged for spindle fiber (Atb2-mCherry) ( Figure 1H ). Nocadazole-treated cells had ablated spindle fibers, leaving only the spindle pole body (Sad1-mCherry) visible, indicating that cells were uniformly arrested in mitotic phase. To test the temperature-sensitive polymerase mutant alleles, we carried out the experiment at 36 ºC. Like *cdc2-asM17 cut9-66* 5 strain, WT cells in *cdc2-asM17 nda3-KM311* background had a significant increase in MMS-treated condition. DNA polymerase alpha catalytic subunit mutant *pol1-1* , DNA polymerase delta catalytic subunit alleles *cdc6-23* and *pold-ts1,* as well as DNA polymerase epsilon catalytic subunit allele *cdc20-M10* strains all failed to increase DNA synthesis in MMS-treated conditions ( Figure 1I ). These results suggest that multiple polymerases contribute to MMS-induced post-replicative DNA synthesis in G2/M.

In summary, this study investigated DNA repair proteins, checkpoint kinases, and polymerases that play a role in DNA synthesis that occurs outside of S-phase in response to replication stress induced by MMS. TLS and BIR repair pathways but not HR repair pathway are largely responsible for MMS-induced G2/M DNA synthesis. It is surprising that mutants from various repair pathways are able to almost fully block post-replicative DNA synthesis. Checkpoint kinases, structure-specific endonucleases Mus81 and Swi10, and exonuclease Exo1 also contribute to post-replicative DNA synthesis. Together these findings suggest that multiple repair pathways and polymerases are involved in promoting DNA synthesis outside of S-phase in response to replication stress. Mechanistical details of how these various repair and checkpoint pathways are linked together remain for future investigations.

## Methods

**Yeast strains and Media**

*Schizosaccharomyces pombe* strains (Table 1) were cultured using standard protocols and media (Sabatinos and Forsburg 2010) , grown in supplemented Edinburgh minimal medium (EMM).

**Table:** 

Strain	Genotype	Source
FY10617	h- cut9-665 cdc2-asM17 leu1-32::hENT1-leu1+(pJAH29) his7-366::hsv-tk-his7+(pJAH31) ura4-D18	(Singh et al. 2021)
FY10645	h- cut9-665 cdc2-asM17 kpa1∆::bleoMX leu1-32::hENT1-leu1+(pJAH29) his7-366::hsv-tk-his7+(pJAH31) ura4-D18	
FY10633	h+ cut9-665 cdc2-asM17 polη (eso1-rad30∆)::kanMX6 (pol-eta deficient) dinB∆::bleMX6 rev3∆::hphMX6 rev1∆::ura4+ leu1-32::hENT1-leu1+(pJAH29) his7-366::hsv-tk-his7+(pJAH31) ura4-D18	(Kai and Wang 2003)
FY10638	h+ cut9-665 cdc2-asM17 pcn1-K164R::ura4 leu1-32::hENT1-leu1+(pJAH29) his7-366::hsv-tk-his7+(pJAH31) ura4-D18	
FY10682	h+ cut9-665 cdc2-asM17 cdc27-D1(160-327) leu1-32::hENT1-leu1+(pJAH29) his7-366::hsv-tk-his7+(pJAH31) ura4-D18	
FY10641	h- smt0? cut9-665 cdc2-asM17 rhp54∆::ura4+ leu1-32::hENT1-leu1+(pJAH29) his7-366::hsv-tk-his7+(pJAH31) ura4-D18	
FY10636	h90 cut9-665 cdc2-asM17 rad22∆::[hisG ura4+ hisG] leu1-32::hENT1-leu1+(pJAH29) his7-366::hsv-tk-his7+(pJAH31) ura4-D18	
FY10637	h+ cut9-665 cdc2-asM17 rad51∆::ura4+ leu1-32::hENT1-leu1+(pJAH29) his7-366::hsv-tk-his7+(pJAH31) ura4-D18	
FY10642	h+ cut9-665 cdc2-asM17 fbh1∆::kanMX leu1-32::hENT1-leu1+(pJAH29) his7-366::hsv-tk-his7+(pJAH31) ura4-D18	
FY10765	h+ rad52-aid-V5-Turg1:kanMX6, arg3::bleMX6-arg3+-Padh1-OsTIR1(F74A)-TADH1 cut9-665 cdc2-asM17 leu1-32::hENT1-leu1+(pJAH29) his7-366::hsv-tk-his7+(pJAH31) ura4-D18	(Watson et al. 2021)
FY10774	h- pku70::kanr cut9-665 cdc2-asM17 leu1-32::hENT1-leu1+(pJAH29) his7-366::hsv-tk-his7+(pJAH31) leu1-32 ura4-D18	
FY10775	h- rhp9∆::ura4+ (=crb2) cut9-665 cdc2-asM17 leu1-32::hENT1-leu1+(pJAH29) his7-366::hsv-tk-his7+(pJAH31) ura4-D18 leu1-32	
FY10681	h- cut9-665 cdc2-asM17 rad3∆::ura4+ leu1-32::hENT1-leu1+(pJAH29) his7-366::hsv-tk-his7+(pJAH31) ura4-D18	
FY10639	h+ cut9-665 cdc2-asM17 chk1∆::ura4+ leu1-32::hENT1-leu1+(pJAH29) his7-366::hsv-tk-his7+(pJAH31) ura4-D18	
FY10683	h- cut9-665 cdc2-asM17 cds1∆::ura4 leu1-32::hENT1-leu1+(pJAH29) his7-366::hsv-tk-his7+(pJAH31) ura4-D18	
FY10631	h+ cut9-665 cdc2-asM17 mus81∆::KanMX leu1-32::hENT1-leu1+(pJAH29) his7-366::hsv-tk-his7+(pJAH31) ura4-D18	
FY10703	h90 cut9-665 cdc2-asM17 rqh1∆:kanMX6-Bioneer leu1-32::hENT1-leu1+(pJAH29) his7-366::hsv-tk-his7+(pJAH31) ura4-D18 leu1-32	
FY10770	h- rad2∆::ura4+ cut9-665 cdc2-asM17 leu1-32::hENT1-leu1+(pJAH29) his7-366::hsv-tk-his7+(pJAH31) leu1-32 ura4-D18	
FY10771	h- rad16∆::ura4 cut9-665 cdc2-asM17 leu1-32::hENT1-leu1+(pJAH29) his7-366::hsv-tk-his7+(pJAH31) ade6-? leu1-32 ura4-D18	
FY10776	h- swi10∆::kanMX cut9-665 cdc2-asM17 leu1-32::hENT1-leu1+(pJAH29) his7-366::hsv-tk-his7+(pJAH31) ura4-D18 leu1-32	
FY10777	h90 exo1∆::ura4+ cut9-665 cdc2-asM17 leu1-32::[hENT leu1+] his7-377::[hsv-tk his7+] ura4-D18	
FY10587	h- cdc2-asM17 sad1-mCherry::Ura4+ kanR<<Pnmt41-mCherry-atb2+ arg3+::ccr1N-GFP(D817 aa1-275))::his5+ ura4-D18	
FY10549	h+ nda3-KM311 cdc2-asM17 leu1-32::hENT1-leu1+(pJAH29) his7-366::hsv-tk-his7+(pJAH31) ura4-D18	
FY10671	h+ pol1-1 nda3-KM311 cdc2-asM17 leu1-32::hENT1-leu1+(pJAH29) his7-366::hsv-tk-his7+(pJAH31) ura4-D18	
FY10685	h+ cdc6-23 nda3-KM311 cdc2-asM17 leu1-32::hENT1-leu1+(pJAH29) his7-366::hsv-tk-his7+(pJAH31) ura4-D18	
FY10670	h+ pold-ts1 (cdc6-ts1) nda3-KM311 cdc2-asM17 leu1-32::hENT1-leu1+(pJAH29) his7-366::hsv-tk-his7+(pJAH31) ura4-D18	
FY10669	h+ cdc20-M10 nda3-KM311 cdc2-asM17 leu1-32::hENT1-leu1+(pJAH29) his7-366::hsv-tk-his7+(pJAH31) ura4-D18	

**EdU uptake assay**

Cells grown in asynchronous culture were arrested in G2 by 2 µM 3-Brb-PP1 (TRC, A602985) for 3.5 h with or without 0.001% of methyl methanesulfonate (MMS) and then were washed twice with supplemented media before being placed at 36 ºC or 100 µg/ml nocodazole (Sigma, M1404) for M-arrest. 10 µM 5-Ethynyl-2’-deoxyuridine was added during G2-arrest and M-arrest. 100 nM 5’adamantyl-IAA (TCI Chemicals, A3390) was added at or during G2- or M-arrest. Cells were spun down and resuspended in 70% ethanol and placed in 4 ºC for fixation. Then fixed cells were washed in 1% BSA containing PBS and processed using Click-iT™ EdU Cell Proliferation Kit for Imaging, Alexa Fluor™ 488 dye (ThermoFisher Scientific, cat #10337), following the manufacturer protocol.

**Microscopy**

Cells were placed on 2% agarose pads sealed with VaLaP (1/1/1 [wt/wt/wt] Vaseline/lanolin/paraffin) for live cell imaging. EdU-Click-iT processed samples were suspended in 20 µl of 1% BSA and then transferred to charged slides (Premiere, 9308W) and heat-fixed at 50 ºC for 5 min. Antifade mounting medium (50% glycerol in water with 0.1% p-phenylenediamine dihydrochloride) with 1 µg/ml DAPI (4’,6-diamidino-2-phenylindole) was then added before placing the coverslip for imaging. Images were acquired using a DeltaVision microscope (with softWoRx version 4.1; GE, Issaquah, WA) using a 60x (for live cells) 100x (for fixed cells) lens, solid-state illuminator, and 12-bit CCD camera. Images were deconvolved and maximum intensity projected from seven z-stacks of 0.5 mm with 0.08-0.5 sec exposure time (softWoRX) (Schindelin et al. 2012) .

**Image analysis**

Images of nuclear EdU-488 were analyzed using ImageJ as in (Kim and Forsburg 2023) . Briefly, binary image of the nucleus of each cell was created from DAPI staining and this ROI (region of interest) was used for assessing EdU-488 intensity in the nucleus. The same area size was then translated to cytoplasmic part within the cell for normalization. Nuclear EdU-488 intensity over cytoplasmic intensity was plotted using GraphPad. WT strain shows the data collected from three biological replicates and mutant strains and Rad52-AID strain shows data from a single biological replicate of four or more technical replicates.
